# Audiovisual Moments in Time: A large-scale annotated dataset of audiovisual actions

**DOI:** 10.1371/journal.pone.0301098

**Published:** 2024-04-01

**Authors:** Michael Joannou, Pia Rotshtein, Uta Noppeney

**Affiliations:** 1 Computational Neuroscience and Cognitive Robotics Centre, University of Birmingham, Birmingham, United Kingdom; 2 University of Haifa, Mount Carmel, Haifa, Israel; 3 Donders Institute for Brain, Cognition and Behaviour, Radboud University, Nijmegen, The Netherlands; University of Manitoba, CANADA

## Abstract

We present Audiovisual Moments in Time (AVMIT), a large-scale dataset of audiovisual action events. In an extensive annotation task 11 participants labelled a subset of 3-second audiovisual videos from the Moments in Time dataset (MIT). For each trial, participants assessed whether the labelled audiovisual action event was present and whether it was the most prominent feature of the video. The dataset includes the annotation of 57,177 audiovisual videos, each independently evaluated by 3 of 11 trained participants. From this initial collection, we created a curated test set of 16 distinct action classes, with 60 videos each (960 videos). We also offer 2 sets of pre-computed audiovisual feature embeddings, using VGGish/YamNet for audio data and VGG16/EfficientNetB0 for visual data, thereby lowering the barrier to entry for audiovisual DNN research. We explored the advantages of AVMIT annotations and feature embeddings to improve performance on audiovisual event recognition. A series of 6 Recurrent Neural Networks (RNNs) were trained on either AVMIT-filtered audiovisual events or modality-agnostic events from MIT, and then tested on our audiovisual test set. In all RNNs, top 1 accuracy was increased by 2.71-5.94% by training exclusively on audiovisual events, even outweighing a three-fold increase in training data. Additionally, we introduce the Supervised Audiovisual Correspondence (SAVC) task whereby a classifier must discern whether audio and visual streams correspond to the same action label. We trained 6 RNNs on the SAVC task, with or without AVMIT-filtering, to explore whether AVMIT is helpful for cross-modal learning. In all RNNs, accuracy improved by 2.09-19.16% with AVMIT-filtered data. We anticipate that the newly annotated AVMIT dataset will serve as a valuable resource for research and comparative experiments involving computational models and human participants, specifically when addressing research questions where audiovisual correspondence is of critical importance.

## Introduction

Many events generate auditory and visual signals that evolve dynamically over time. To obtain a more robust and reliable percept of the environment human observers integrate redundant and complementary information across sensory modalities [1]. For instance, audiovisual integration facilitates speech comprehension in noisy and adverse environments [2]. As work in the area of deep learning has progressed, researchers have looked to take advantage of additional information available across multiple modalities to improve recognition performance. In speech recognition, for instance, researchers have developed deep neural networks (DNNs) to leverage audiovisual correspondences [3, 4]. To solve audiovisual speech recognition, DNNs rely on large labelled datasets with high levels of audiovisual correspondence [4, 5].

In the domain of action recognition, audiovisual events produce corresponding audio and visual signals, and these correspondences could be used to improve recognition rates [6]. Despite the improved recognition rates available, annotations for the most popular large action recognition datasets are either visual-only or modality-agnostic (occurring in either/both modalities) [7–11]. This leads to a lack of audiovisual correspondence in available datasets, as an event may have only occurred in a single modality, or the auditory and visual signals may have accurately represented the labelled action despite being generated by different events.

Although the majority of action recognition datasets are not annotated for audiovisual events (an event with both an auditory and visual signal) [7–11], some researchers have begun to target the audiovisual domain in their data collection/annotation. [12] carried out a large-scale annotation task that assessed whether an event is present in both the audio and visual streams. But this annotation scheme only ensured that audio and visual signals corresponded to the label, not that they were caused by the same event. [13] released the Audio-Visual Event Dataset (AVE) of 4,143 audiovisual event videos, but videos can be up to 10 seconds long and are only confirmed to have at least 2 seconds of the labelled audiovisual action, with only 66.4% of videos containing the labelled audiovisual action throughout their duration. Similarly, [14] produced the Look, Listen and Parse Dataset of 11,849 YouTube video clips. But the train set again contains 10 second videos and is only confirmed to have audio/visual events for 1 second or more, with only the test set containing more fine-grained audiovisual labels. Another audiovisual action recognition dataset is Epic-Kitchens [15] with videos depicting egocentric (1^st^ person) hand object interactions in kitchens. But the deep learning community still lacks a high quality allocentric (3^rd^ person) audiovisual action dataset.

To facilitate deep learning research in the audiovisual domain, we present Audiovisual Moments in Time (AVMIT), a set of 57,177 audiovisual annotations for the Moments in Time dataset (MIT) [9]. To obtain AVMIT, we take a subset of the MIT dataset and run a large-scale annotation regime. Growing research reveals noncompliance [16, 17] of participants on Amazon Mechanical Turk [18], including [19] were 49% of turkers were found not to be wearing headphones despite reporting they did. To ensure high quality annotations, we elected to train raters and have them perform the task in a controlled lab setting. AVMIT contains 3 independent participant ratings for 57,177 videos (171,630 annotations). We further screened MIT videos to select a highly controlled audiovisual test set of 960 videos across 16 action classes, named the AVMIT test set. The AVMIT test set is suitable for human and DNN experimentation, particularly for studies concerned with audiovisual correspondence. Finally, to lower the computational requirements to train DNNs on audiovisual problems, we provide two sets of audiovisual embeddings that can be used to further train audiovisual DNNs. To obtain each set of audiovisual embeddings, we use convolutional neural networks (CNNs); VGGish [20] (audio) and VGG-16 [21] (visual) or YamNet [22] (audio) and EfficientNetB0 [23] (visual) and extract features from all AVMIT annotated videos.

Beyond building audiovisual recognition models, AVMIT can be used for audiovisual separation [24] (using audiovisual information to separate sounds from different sources), audiovisual localisation [13, 24–26] (finding the sound source in the visual context), audiovisual correspondence learning [25, 27] (discerning if the audio and visual signal emanated from the same source/type of source), audiovisual synchronization learning [24, 28] (detecting misalignments between audio and visual streams), audiovisual parsing [14, 29] (parsing a video into temporal event segments and labelling them as either audible, visible, or both) and audiovisual generation [12, 30] (generating audio from visual or visual from audio) and any other tasks that exists only in the audiovisual domain. Further, AVMIT serves as a valuable resource for research and comparative experiments involving computational models and human observers that are known to rely on audiovisual correspondences [1]. As DNNs are now commonly used as predictive models of human behaviour in vision [31] and audition [19], AVMIT supports this research to take a step into the audiovisual domain.

## Methods

### Participants

To rate the videos, eleven participants (10 females; mean age 26.18, range 19-63 years) were recruited over the period starting 6^th^ September 2018 and ending 22^th^ May 2019. Participants were first asked to complete a safety questionnaire and provided with an instruction sheet. Instructions were further explained verbally before participants gave informed, written consent to take part in the experiment. No participants were excluded. Each participant annotated a subset of the candidate videos. All reported normal hearing and normal or corrected-to-normal vision. Participants were reimbursed for their participation in the task at a rate of £6 per hour, plus a bonus of 10p paid for correct classification of randomly interspersed ground truths (further detailed in the Bonus Section). Participants on average earned a total (hourly payment + bonus) of less than £7 per hour. The research was approved by the University of Birmingham Ethical Review Committee.

### Annotation workspace

Participants were seated at a desk in an experiment cubicle or quiet area to complete this task. The experiment was presented on a Dell Latitude 5580 laptop with 15.6” screen and Linux Ubuntu 18.04.2 LTS operating system. Auditory stimuli were presented via a pair of Sennheiser HD 280 Professional over-ear headphones. The experiment was programmed in Python 2 [32] and Psychopy 2020.2.10 [33].

### Selection of MIT videos

Prior to the annotation task, we carried out a selection process to obtain a subset of MIT videos that were more likely to contain audiovisual actions. We first obtained the labelled training (802,264 videos) and validation (33,900 videos) sets of the MIT dataset. The events depicted in these videos unfold over 3 seconds. For many of the classes in the MIT dataset, audio data would not help recognition of the labelled event (e.g. “imitating”, “knitting”, “measuring”). We carefully curated a subset of 41 audiovisual classes (corresponding to 88,579 training videos and 4,100 validation videos) that offer a wealth of informative audio and visual correspondences, enabling enhanced classification through the integration of these signals.

To increase the number of videos in our selected AVMIT classes, we obtained videos from similar, but excluded, MIT classes, relabelled them, and added them to our annotation task. Incorrectly relabelled videos would be annotated by our participants as not containing the labelled audiovisual event. Table 1 displays those AVMIT classes alongside the other MIT classes that were relabelled and added to the annotation task. To ensure that candidate videos included audio and video components, we removed videos without audio streams or whose amplitude did not exceed 0 (digital silence).

**Table 1:** Relabelled MIT classes.

AVMIT class	Additional MIT class
Giggling	Laughing
Frying	Cooking, Boiling
Inflating	Blowing
Pouring	Spilling, Drenching, Filling
Diving	Swimming, Splashing
Raining	Dripping

Excluded MIT classes that were relabelled and added to the annotation task.

### Annotation procedure

Next, we created a video annotation task that could be carried out by multiple trained participants to identify if videos contained the labelled audiovisual event and whether it was the most prominent feature. This procedure was similar to the annotation procedure carried out in [12] to produce the VEGAS dataset.

Participants were presented with a series of audiovisual videos and were instructed to provide a button response after each had finished playing. On each trial, participants were presented with a 3 second video and then classified it as 1:“unclean”, 2:“moderately clean” or 3:“very clean”. To provide a classification, participants were trained to use the following logic:

1. Was the labelled audiovisual event present?:
  No: give a 1 rating
  Yes: move to the next question
2. Was the labelled audiovisual event the most prominent feature?:
  No: give a 2 rating
  Yes: give a 3 rating

For this task, an event was considered to be the most prominent feature if it was of longer duration and higher intensity than any other event in the same video. Intensity related to amplitude of event audio and size of the event’s region of interest. Each video was rated by at least 3 participants.

**Fig 1:**
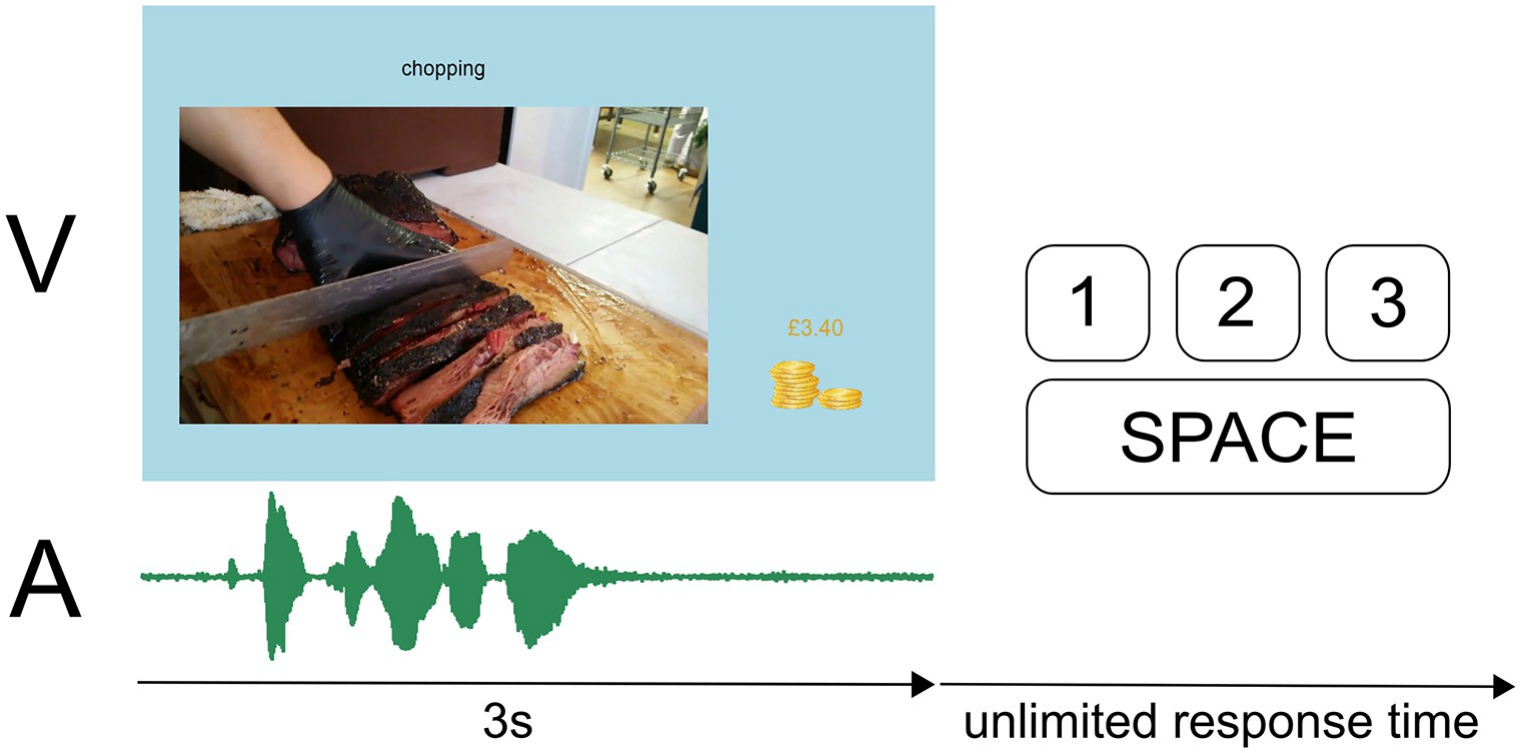
Annotation task schematic. Task screen displays a chopping video with label and accumulated bonus. Video plays for 3 seconds alongside audio stimuli. Participants watched and listened to the audiovisual video before providing a rating.

During video presentation, the screen displayed the suggested action label at the top, the video in the bottom-left (videos had different resolutions so they were each given a common left edge position and bottom edge position) and a bonus counter in the bottom right (Fig 1). Together with the video, participants were presented with the audio via headphones. After the video and audio stopped playing, the program waited until the participant pressed a key. The options were; 1, 2, 3, space, where the numbers referred to the classification system described above and the space key would replay the video. Participants were able to replay the video and audio any number of times they like before making a classification. If the participant made a classification while the video was still playing, a warning screen would fill the display, instructing the participant not to press a key too early. This was particularly important given that the audiovisual video content after an early classification may change the answer to question 2. After a classification was made, the bonus counter would be updated, and the new label title and audiovisual video would appear.

### Quality control

In order to ensure the quality of the AVMIT dataset, we opted to use trained participants in a controlled environment rather than Amazon Mechanical Turk. Participants were required to complete the training exercise, before they could participate in the annotation task. Before starting, each participant was given a set of instructions that outlined the task on a sheet of paper. These instructions were then verbally explained to them. The participants then undertook a training exercise whereby a video from each class was presented and the possible classification and reasoning was discussed with the author (MJ) of the study. The participants were then screened to ensure that they understood the task by classifying another set of videos (1 video per class) under the observation of the author. Of these videos, the participants needed to classify 38 of the 41 videos according to the author’s ground truth. Of the 11 participants that completed the training and testing exercise, all participants passed and went on to take part in the annotation task.

Another strategy we employed, was to provide bonus payments to participants in order to ensure engagement and provide positive feedback. A bonus payment of 10p (GBP) was given for each classification of a video for which a ground truth was available. To obtain ground truths, 2,000 videos were uniformly sampled from the set of candidate videos prior to the annotation task and then classified by one of the authors (MJ). These audiovisual videos were distributed throughout the annotation task and participants were unaware of the possibility of a bonus when completing a trial. If the participant gave a matching classification for one of these previously classified audiovisual videos, they would receive a bonus, which was added to their total in the bottom right of the screen (Fig 1). This bonus accumulated over their sessions and was paid at the end of participation alongside their hourly compensation.

Quality of annotations was further ensured by using at least 3 participants to rate each video, in line with the procedure of other large dataset annotation schemes [9, 10, 12]. As the AVMIT annotation scheme was run using videos from an existing dataset, AVMIT benefits from the quality assurances of two cleaning processes.

### Test set

**Fig 2:**
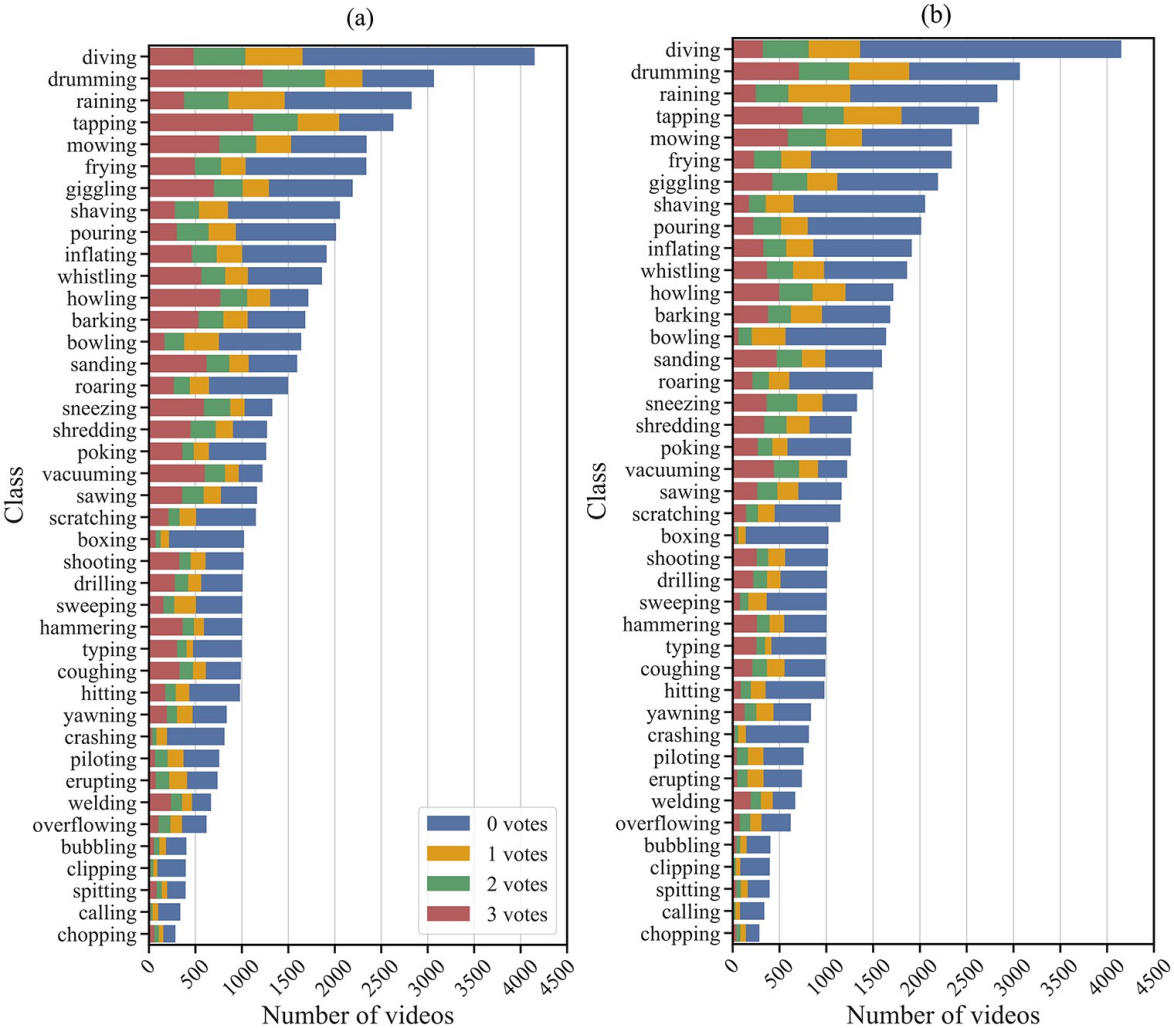
AVMIT annotations. Number of MIT videos in each class that obtained a ‘yes’ vote from 0,1,2 or 3 participants when asked the following questions: (a) Was the labelled audiovisual event present? (b) Was the labelled audiovisual event the most prominent feature?.

We ran further screening to obtain a highly controllable test set for human and deep neural network experiments. This process was 2 stages; class filtering and video filtering. Many classes did not contain a sufficient number of clean audiovisual videos for training and testing a deep neural network (Fig 2). We used a majority vote criteria to obtain those videos containing the labelled audiovisual event as a prominent feature. Classes with 500 or more videos that meet this criteria were accepted into the test set. Just 16 of 41 classes met this criteria, although this is in line with test sets in the humans vs. DNN literature [34]. With test classes obtained, we then applied video filtering. In order to ensure reliability, we set as a criterion that all participants must agree that the audiovisual event was present and the key feature of the video. In order to ensure a level of homogeneity in the dataset, we obtained those audiovisual videos with a visual frame rate of 30fps and further cleaned them, removing videos that:

- Had been edited to appear as though something supernatural had occurred (such as something appearing or disappearing instantaneously)
- Had an excessive number of time-lapses
- Contained frames with excessive watermarks or writing on the frames
- Consisted of 2 video streams
- Were not naturalistic (depicting cartoons or simulations)

From the subset of filtered videos, 60 videos were uniformly sampled from each class and used to provide the AVMIT test set (60 videos per class, 16 classes, 960 video test set). By comparison, naturalistic stimuli sets for human experiments in the area of psychology and neuroscience often have far fewer stimuli [6, 35, 36] and these may be further manipulated according to a variety of conditions to effectively multiply test set size. After filtering train videos with AVMIT in our experiments, this test set formed approximately 12% of our total samples.

### Neural network embeddings

We created 2 sets of audiovisual embeddings; those obtained using VGGish [20] and VGG-16 [21] and a second set obtained using YamNet [22] and EfficientNetB0 [23]. Both VGG-16 and EfficientNetB0 were trained on ImageNet [37] and VGGish and YamNet were trained on AudioSet [38]. Prior to feature extraction by these CNN models, audio and visual data was preprocessed.

If the audio was stereophonic rather than monophonic, a monophonic stream was obtained using pydub.AudioSegment.set_channels [39], taking the mean of the left and right channels (Eq 1). Where S_new_ is the new monophonic audio sample, S_L_ is the original left sample and S_R_ is the original right sample.

$$\begin{array}{c} S_{\text{new}}=0.5\cdot S_{L}+0.5\cdot S_{R} \end{array}$$ (1)

Audio data of a depth other than 16 bits was cast to 16 bits using pydub.AudioSegment.set_sample_width [39]. These int16 audio samples were then mapped from the range [-32768, 32767] (2^15^ with one bit dedicated to sign) to the range [-1.0, 1.0] by dividing by the maximum value of 32768.0. The audio was then resampled to 16 kHz before spectrograms were calculated.

Next we carried out a short-time Fourier transform (STFT) to provide a frequency decomposition over time. We used a frame size of 25ms (the period over which signals are assumed to be stationary) and a 10ms stride (the frequency with which we obtain a frame). Overlapping frames help to ensure that any frequency in the signal that may exist between otherwise non-overlapping frames are captured in the spectrum. A Hann filter was applied to each of the frames before a fast Fourier transform (FFT) was carried out. A log mel spectrogram was then obtained using a mel filter bank of 64 filters, over the range 125-7500 Hz, and then finding the logarithm of each spectrum (plus a small delta of 0.01 to avoid taking the log of 0; Eq 2).

$$\begin{array}{c} \text{log}\;\text{mel}\;\text{spectrogram}=\text{log}(\text{mel}\;\text{spectrogram}+0.01) \end{array}$$ (2)

The log mel spectrograms were windowed into smaller 960ms spectrograms, ready for the CNN. Audio preprocessing deviated between the VGGish and YamNet embeddings in this final stage of preprocessing in accordance with their training regimes [20, 22]. For VGGish, the stride was 960ms between windows, for YamNet, the stride was 480ms.

For visual processing, we sampled frames according to the frequency of the complementary audio features; 960ms for VGG-16 and 480ms for EfficientNetB0. This was to provide a similar number of audio and visual embeddings per sample. Frames were then resized to dimensions of 224x224x3 using OpenCV [40] in line with the expected input size of the CNN models. For VGGish the images were then zero centred, but for EfficientNetB0, images were rescaled, normalised and then zero-padded.

## Dataset statistics

The focus of the AVMIT project was to provide a large, annotated audiovisual action dataset to facilitate the training of deep neural networks in the audiovisual domain. AVMIT contains annotations for 57,177 videos (171,630 annotations; Fig 2) that can be used for training deep neural networks where audiovisual correspondence is key. AVMIT is confirmed to contain 23,160 videos (19.3 hours) of labelled audiovisual actions, of which 17,891 (14.9 hours) are the prominent feature of the video, according to majority participant vote. These annotations also provide insight into the quality of MIT labels in the audiovisual domain. For instance, the majority of MIT videos were confirmed to not feature the audiovisual action described by the label (Fig 2a).

Motivated to better understand the quality of AVMIT annotations, we sought to quantify the audiovisual correspondence of the annotated videos. For this, we employed the multimodal versatile network (MMV) from [41] as a method to measure the similarity of a video’s audio and visual stream.

**Fig 3:**
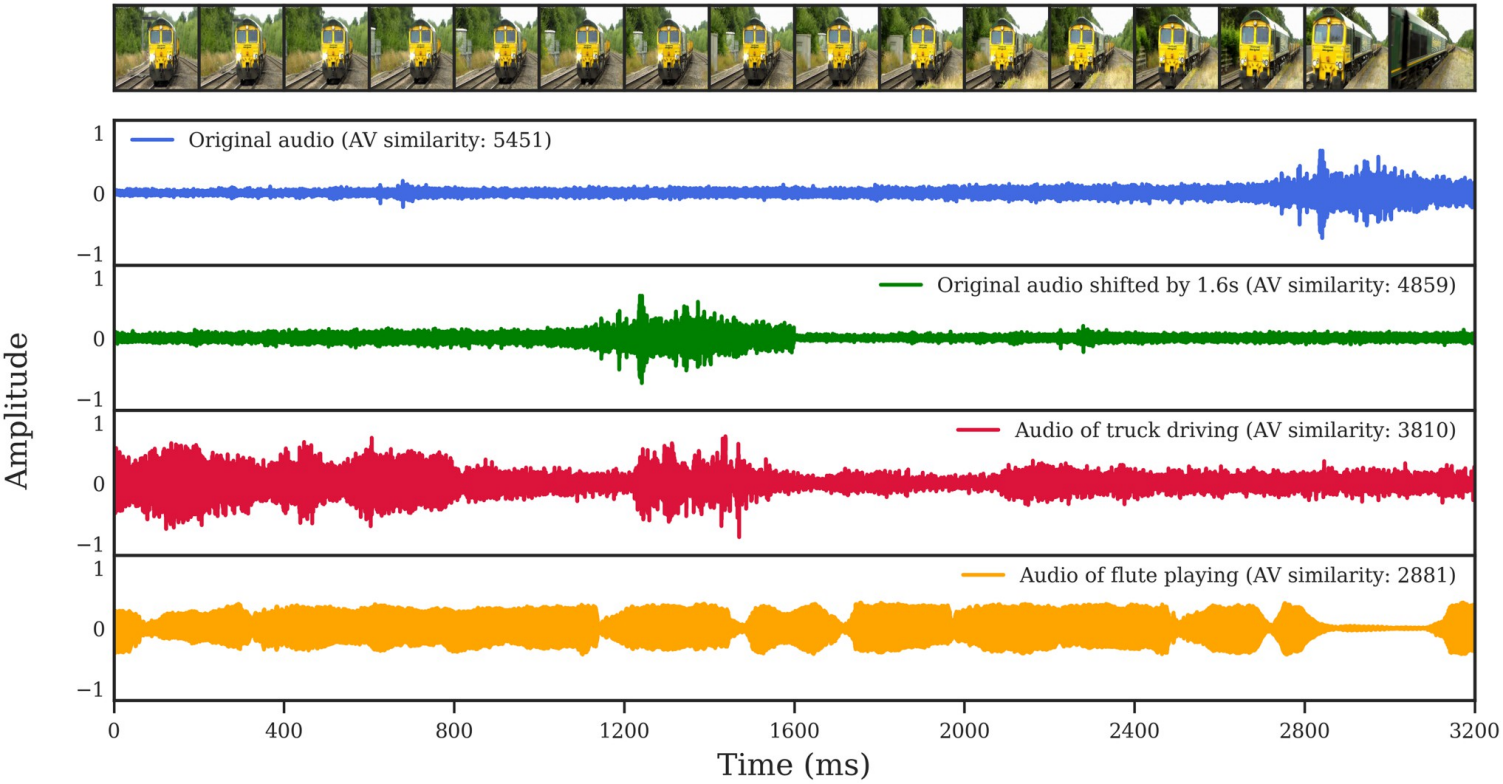
Examples of MMV audiovisual similarity estimates. Visual frames from a video (above) with 4 possible audio streams below. Each audio stream has a corresponding audiovisual (AV) similarity score, estimated by MMV, when combined with the visual stream. The original audio stream leads to the highest AV similarity, which is decreased by introducing temporal asynchrony (shifting audio 1.6 seconds). Increasing the semantic distance between the audio and visual stream further decreases the AV similarity (from ‘train’ to ‘vehicle’ to ‘instrument’).

MMV is trained to project audio and visual signals onto a common embedding space where cross-modal comparisons can be made. The multimodal contrastive loss used to train MMV causes co-occurring audio and visual signals from a video to be similar in embedding space, and signals from different videos to be dissimilar. The audiovisual similarity is calculated by taking the dot product of the audio and visual embedding [41]. Thus the audiovisual similarity reported as part of our analysis is an MMV estimate of the likelihood of co-occurrence in a video, as described in [41]. A large similarity score indicates that the audio and visual signals co-occurred, a low (or negative) similarity score indicates that they are less likely to have co-occurred and may pertain to different events (Fig 3).

**Fig 4:**
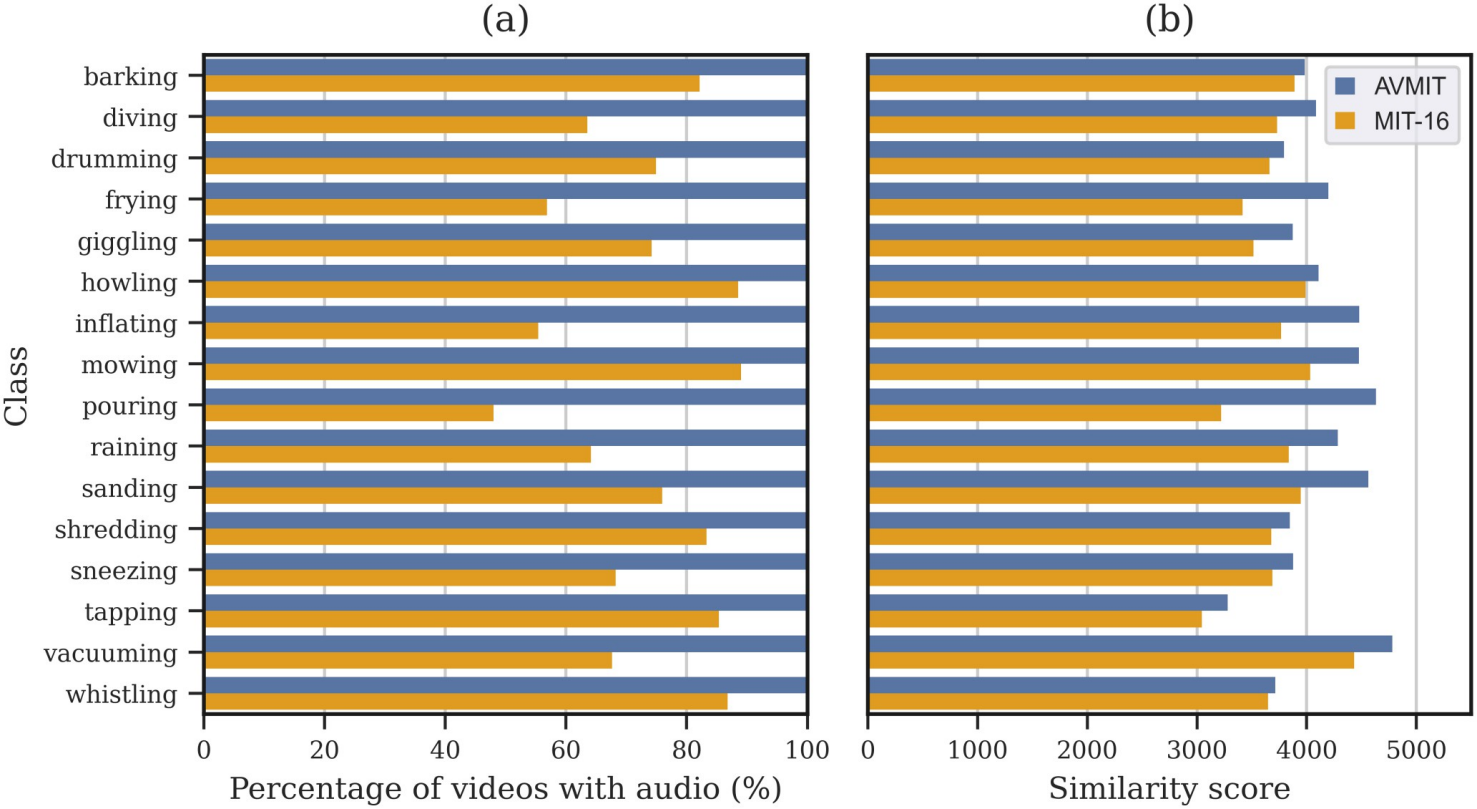
AVMIT vs MIT-16. Comparison of MIT videos, corresponding to 16 AVMIT test classes, before and after filtering with AVMIT annotations. Filtering retained only videos containing the audiovisual event as a prominent feature, according to the majority of participants. (a) Shows the percentage of MIT-16 and AVMIT videos with an audio stream across each class (b) Shows the average similarity score, as estimated by MMV, for AVMIT and MIT-16 across 16 classes.

First, we considered the utility of AVMIT annotations by measuring the audiovisual similarity before and after they were used for filtering. For this we prepared a dataset, MIT-16, containing all original MIT videos from the 16 AVMIT test classes. We then used the AVMIT annotations to retain only those videos rated as containing the audiovisual event as a prominent feature by the majority of participants. Those MIT-16 videos without audio information were removed for this analysis (Fig 4a) and the audiovisual similarity was estimated only on those videos with both audio and visual streams. The average audiovisual similarity score, as estimated by MMV, is higher for AVMIT-filtered videos across all classes (Fig 4b). This indicates that AVMIT annotations of prominent audiovisual actions correspond to higher proximity in MMV embedding space (are more similar).

**Fig 5:**
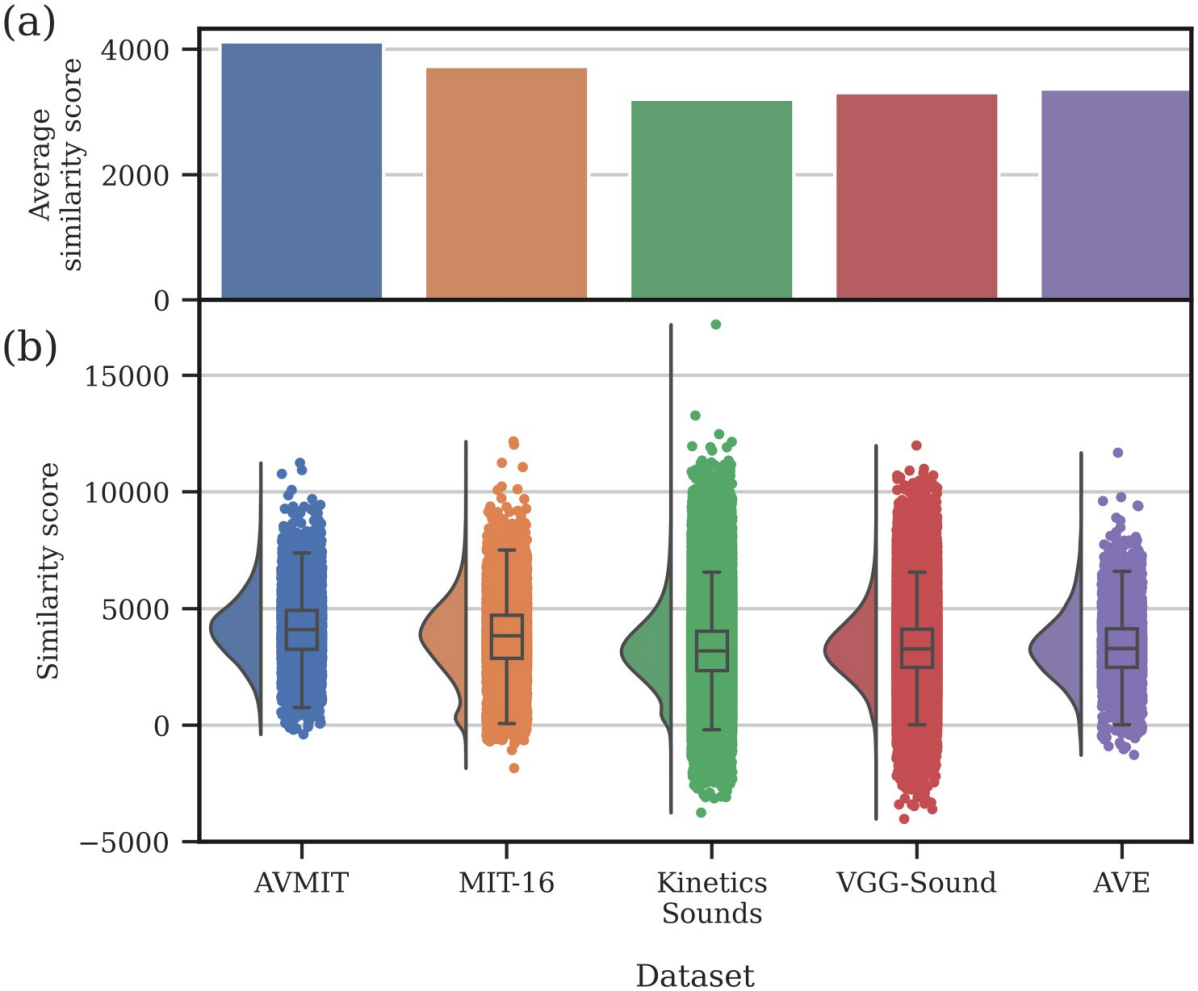
AVMIT vs other datasets. Audiovisual similarity scores, as estimated by MMV, across a series of audiovisual action recognition datasets; AVMIT (ours), MIT-16, Kinetics-Sounds, VGG-Sound and AVE. (a) Average audiovisual similarity score across entire datasets. (b) Rain cloud plot showing the distribution of audiovisual similarity scores for each dataset.

To further consider how AVMIT and MIT-16 compare to other popular audiovisual action datasets in the literature, we used MMV to measure their audiovisual similarity (Fig 5). We ran this analysis on Kinetics-Sounds [27], VGG-Sound [42] and AVE [13], finding AVMIT to have a considerably higher average audiovisual similarity score (Fig 5a). We also find the distribution of audiovisual similarity scores across AVMIT to be superior to other measured datasets, with far fewer videos that have highly dissimilar audiovisual content (Fig 5b).

Whilst the audiovisual similarity scores provided by MMV demonstrate the utility of AVMIT against other popular datasets, we further outline AVMIT’s place in the literature in Table 2. AVMIT is the only large, annotated audiovisual action dataset to our knowledge to provide a controlled audiovisual test set appropriate for human experiments. AVMIT is also the largest audiovisual action dataset annotated with trained participants. We include Epic-Kitchens [15] in Table 2 as a large audiovisual action dataset, although it is egocentric and so not directly comparable.

**Table 2:** Statistics of popular audiovisual action datasets.

Dataset	Year	Controlled Test Set	Annotation Modality	Trained In-house Annotators	Perspective	Hours	Videos
AVMIT	2023	True	Audiovisual	True	Allocentric	48	57,177
AVE	2018	False	Audiovisual	True	Allocentric	12	4,143
EPIC-KITCHENS-100	2021	False	Audiovisual	False	Egocentric	100	700
Kinetics-Sounds	2017	False	Modality-Agnostic	False	Allocentric	556	20,000
VGG-Sound	2020	False	Audio	False	Allocentric	560	200,000

## Data description

AVMIT consists of 4 components; audiovisual annotations of 57,177 MIT videos, a selection of 960 MIT videos designated as the AVMIT test set and 2 sets of audiovisual feature embeddings. All of these are available at https://zenodo.org/record/8253350.

The AVMIT annotations are available in the file named video_ratings.csv. Each row in the csv file corresponds to a video (containing all corresponding ratings from participants). Each video was rated 3 times. Videos rated less than 3 times were removed. The video_ratings.csv fields are described in Table 3. The annotations are visualised in Fig 2. The test set details are provided in test_set.csv, fields are described in Table 4.

**Table 3:** Description of data in video_ratings.csv.

Field	Description
filename	“MIT class subdirectory/ video name”
r1	number of ‘1’ ratings given
r2	number of ‘2’ ratings given
r3	number of ‘3’ ratings given
AVMIT_label	as displayed to participants in annotation task
MIT_label	original dataset label
video_location	training or validation directories of MIT
tfrecord_filename	subdirectory and filename of corresponding audiovisual feature embeddings

**Table 4:** Description of data in test_set.csv.

Field	Description
filename	“MIT class subdirectory/ video name”
AVMIT_label	as displayed to participants in annotation task
MIT_label	original dataset label
video_location	training or validation directories of MIT
new_filename	“AVMIT label subdirectory/ new video name”
tfrecord_filename	subdirectory and filename of corresponding audiovisual feature embeddings

There are 2 archived feature embedding directories; AVMIT_VGGish_VGG16.tar contains the audiovisual embeddings, extracted by VGGish (audio) and VGG-16 (visual) for all AVMIT videos, AVMIT_YamNet_EffNetB0.tar contains the audiovisual embeddings extracted by YamNet (audio) and EfficientNetB0 (visual) for all AVMIT videos. Both sets of feature embeddings have the same directory structure, containing 1 subdirectory per action class (e.g. ‘barking’) for all 41 classes. Inside each class sub-directory lies a.tfrecord file for each AVMIT video. Each tfrecord contains a number of context features; filename, label, number of audio timesteps, number of visual timesteps and 2 sequence features; audio data and visual data. For YamNet-EffNetB0 embeddings, audio data has dimensions (timesteps, 1,024) and visual data has dimensions (timesteps, 1,280). For VGGish-VGG16 embeddings, audio data has dimensions (timesteps, 128) and visual data has dimensions (timesteps, 512).

## Usage notes

AVMIT is available at https://zenodo.org/record/8253350. To use the audiovisual feature embeddings, provided as part of this work, directly. An example python script, feature_extractor/read_tfrecords.py, is provided at https://github.com/mjoannou/audiovisual-moments-in-time to demonstrate how to read these tfrecords into a tensorflow.data.Dataset. AVMIT annotations in video_ratings.csv can be used to filter these embeddings for audiovisual content, and test_set.csv can be used to identify those embeddings intended for testing.

To use raw videos, one needs to download the well-established Moments in Time dataset by visiting http://moments.csail.mit.edu/ and fill out a form before access to the dataset is sent via email. Once access to the MIT dataset is granted, AVMIT annotations, available in video_ratings.csv, can be used to filter videos according to audiovisual content prior to training computational models. The AVMIT test set can also be used alongside the MIT videos, identifying 960 videos suitable for testing computation models and human participants alike. If one wishes to extract tfrecords, in a similar manner to our work, this is demonstrated in feature_extractor/extract_features.py.

## Experiments

### Train sets

Two train sets were prepared for both of our experiments; an audiovisual train set using AVMIT annotations and a larger modality-agnostic (audio and/or visual) train set of MIT embeddings named MIT-16. Both train sets contained embeddings corresponding to the 16 AVMIT test set classes. To prepare the audiovisual train set using AVMIT annotations, we obtained only those embeddings that contained the labelled audiovisual event as a prominent feature, according to majority participant vote. To construct the second train set, all MIT embeddings corresponding to the 16 AVMIT classes were obtained. Embeddings corresponding to the AVMIT test were then removed from both train sets. Finally, the number of train embeddings across each class was balanced by sampling the maximum possible number of embeddings (AVMIT: 456 per class, MIT-16: 1,406 per class).

### Experiment 1: Audiovisual action recognition

#### Outline

Increased statistical similarity between train and test set leads to increased test set performance for DNNs. We assert that in order to obtain a model with high audiovisual action recognition performance, one should optimise DNNs on audiovisual action recognition rather than modality-agnostic action recognition. In this way, DNNs may learn to better leverage audiovisual correspondences.

In this experiment we explored the performance benefits associated with training on purely audiovisual actions using AVMIT annotations. We created a series of DNNs and trained one instance on MIT-16 (modality-agnostic data) and another instance on AVMIT-filtered data. Each trained model was then tested on an audiovisual action recognition test set; the AVMIT test set of audiovisual action events. This is a similar protocol to [14] in that we use a carefully curated test set. We hypothesised that AVMIT models would obtain higher audiovisual action recognition rates.

#### DNN architectures

Each architecture effectively consisted of a (frozen) AudioSet-trained CNN, a (frozen) ImageNet-trained CNN, some shared (trainable) audiovisual operations followed by a (trainable) RNN. For the CNNs, architectures either used VGGish [20] (audio) and VGG-16 [21] (visual) or YamNet [22] (audio) and EfficientNetB0 [23] (visual). Although practically, we provide these embeddings as part of this work and we trained on them directly. These architectures allowed us to leverage powerful pretrained unimodal representations but ensure that any learnt audiovisual features would arise from training on AVMIT/MIT-16 alone. We select similar architectures in each set of embeddings to help prevent overpowered unimodal representations in the trained classifiers and ensure both auditory and visual embeddings are useful.

**Fig 6:**
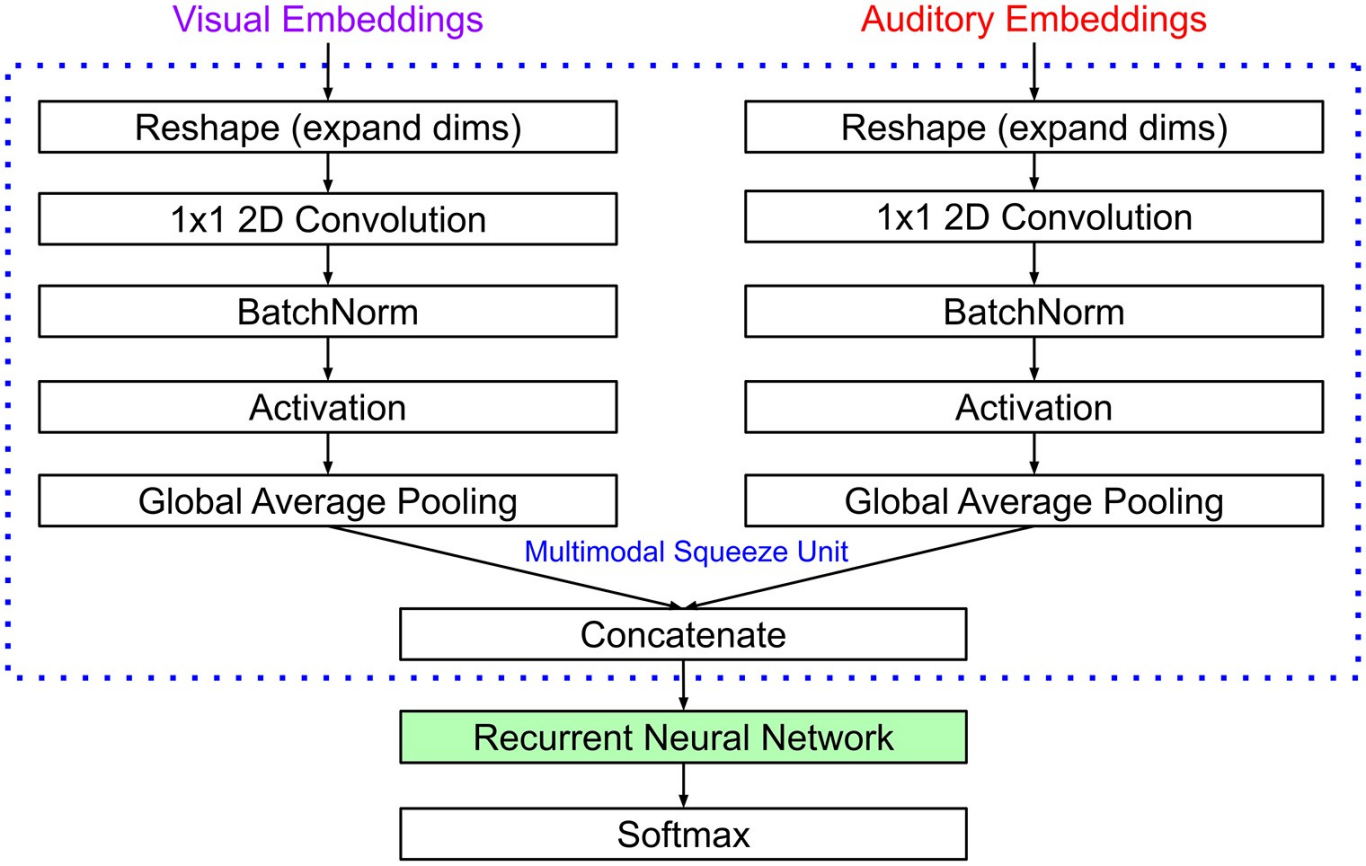
Audiovisual action recognition task architecture. Processing stream for the audiovisual action recognition task. Audio and visual representations are transformed to be the same size and concatenated at each timestep before processing with an RNN and softmax layer.

As the audio and visual embeddings are of different sizes, we added batch-norm convolutional layers and global average pooling operations to each, individually, prior to concatenation. We refer to this series of processes as a multimodal squeeze unit (Fig 6). This is to ensure that there are an equal number of RNN connections dedicated to the processing of auditory and visual information. Following the multimodal squeeze unit, was one of three well-known RNN architectures; fully-recurrent neural network (FRNN, also known as a ‘basic’ or ‘vanilla’ RNN), gated recurrent unit [43] or a long short-term memory unit [44].

#### Hyperparameter search and training

We ran a hyperparameter search (random search) on each embedding-set/RNN combination with the MIT-16 dataset. To use MIT-16 for both the train and test set of the hyperparameter search, we elected to use the bootstrap method; sampling the train set from MIT-16 with replacement, and evaluating it on the out-of-bag (OOB) samples. By optimising the hyperparameters on the MIT-16 dataset, we biased the experiment in favour of MIT-16 trained RNNs, thus strengthening any observed AVMIT related performance gains. For each embedding-set/RNN combination (2 x 3 = 6), we created 300 surrogate models, each with a particular combination of hyperparameter values that were uniformly sampled from the hyperparameter sets or intervals.

We searched over the following hyperparameters; number of filters, n_bottleneck_, in the audiovisual bottleneck (1x1 2D Convolution) where n_bottleneck_ ∈ {32, 64, 128, 256}, the activation function, a, of the audiovisual bottleneck, where a ∈ {relu, swish}, the number of Recurrent Neural Network units, n_RNN_, where n_RNN_ ∈ {32, 64, 128, 256}, the dropout rate, d, for the RNN, where d ∈ {0.1, 0.2, 0.3, 0.4, 0.5} and the learning rate, l, of the model, where l ∈ [1.0x10^−5^, 5.0x10^−3^]. During the random search, RNNs were trained in the same manner (Adam optimiser [45] and exponential learning rate decay) as during final training, the only exception being that early stopping was reduced from 20 epochs to 8 in order to save time during the random search. The best performing configurations for each RNN (Table 5) were selected for comparison across all experiments.

**Table 5:** Hyperparameter search results: Selected hyperparameters.

Embeddings	RNN	RNN Units	Bottle Units	Act.	Dropout	LR	Trainable Params
YamNet + EffNetB0	FRNN	128	256	swish	0.3	7.05 x 10^-5^	675,472
YamNet + EffNetB0	GRU	128	64	swish	0.5	7.25 x 10^-5^	248,976
YamNet + EffNetB0	LSTM	64	256	swish	0.3	4.10 x 10^-5^	740,112
VGGish + VGG-16	FRNN	256	256	swish	0.4	1.05 x 10^-4^	366,352
VGGish + VGG-16	GRU	128	256	relu	0.5	3.92 x 10^-4^	413,968
VGGish + VGG-16	LSTM	256	256	swish	0.5	1.74 x 10^-4^	956,944

For each hyperparameter combination, we trained one RNN instance (row in Table 5) on AVMIT, and another instance on MIT-16. The cross-entropy loss function was used as a measure of loss, and the RNN was trained with backpropagation and the Adam optimiser [45]. Each RNN was trained for up to 200 epochs with a batch size of 16 samples, although with an early stopping of 20 epochs, all RNNs executed training before that point. All learned parameters were then fixed in place throughout testing.

#### Evaluation method

The AVMIT controlled test set was used for testing. As the test set had been well filtered to include only prominent audiovisual events, any learnt audiovisual features should be beneficial to performance. The loss, top 1 classification accuracy (the proportion of trials in which the model gave the highest probability to the correct action class) and the top 5 classification accuracy (the proportion of trials in which the correct action class was assigned one of the top five probabilities) was used to measure performance on this set.

#### Results

All models obtained a top 5 classification accuracy of approximately 100%. Models trained on AVMIT obtained a lower loss and higher top 1 accuracy than their MIT-16 trained counterpart in all cases (Table 6). This result indicates that training a DNN exclusively on audiovisual action events is beneficial for audiovisual action recognition, even outweighing a three-fold increase in training data (additional audio or visual events). A final observation is that the YamNet+EfficientNet-B0 embeddings consistently provided higher performances than VGGish+VGG-16 embeddings.

**Table 6:** Action recognition performance.

Training Set	Embeddings	RNN	Loss	Top 1 Acc. (%)	Top 5 Acc. (%)
AVMIT	YamNet + EffNetB0	FRNN	0.1841	94.58	99.90
MIT 16	YamNet + EffNetB0	FRNN	0.2973	89.79	99.90
AVMIT	YamNet + EffNetB0	GRU	0.1600	95.73	99.90
MIT 16	YamNet + EffNetB0	GRU	0.2430	92.29	99.90
AVMIT	YamNet + EffNetB0	LSTM	0.1674	95.52	99.79
MIT 16	YamNet + EffNetB0	LSTM	0.2366	92.81	100
AVMIT	VGGish + VGG-16	FRNN	0.2980	90.73	99.79
MIT 16	VGGish + VGG-16	FRNN	0.4388	84.79	99.58
AVMIT	VGGish + VGG-16	GRU	0.2917	91.04	99.79
MIT 16	VGGish + VGG-16	GRU	0.4108	85.83	99.69
AVMIT	VGGish + VGG-16	LSTM	0.2892	90.94	99.90
MIT 16	VGGish + VGG-16	LSTM	0.3527	86.98	99.90

### Experiment 2: Supervised Audiovisual Correspondence

#### Outline

In the previous experiment, we showed that higher audiovisual action recognition rates can be achieved by training exclusively on audiovisual events (AVMIT), rather than a larger set of modality-agnostic events (MIT-16). Next, we enquired whether DNNs could more effectively learn about audiovisual correspondences with AVMIT than MIT-16. We hypothesised that this would indeed be the case, due to AVMIT’s high levels of audiovisual similarity (Figs 4b and 5) and high quality audiovisual annotations (Fig 2).

To investigate, we explicitly trained a series of DNNs on an audiovisual correspondence task. Thus far, the unsupervised audiovisual correspondence (UAVC) task has been introduced in the literature as a means to utilise unlabelled/poorly labelled audiovisual data [25, 27]. The UAVC task is a binary classification task that requires the classifier to detect whether a video has intact audiovisual data (corresponds) or if the audio stream has been shuffled between videos (does not correspond). As annotations are not used in this task, audio streams could be shuffled between videos of the same action class and still be considered “non-corresponding”. We introduce the supervised audiovisual correspondence (SAVC) task, whereby audio and visual streams are sampled from the same action class (corresponds) or different action classes (does not correspond). In this way, a “corresponding” video contains an audio stream and a visual stream from the same action class, but not necessarily the same video. This requires that the classifier learn about semantic correspondence only, without temporal correspondence. The SAVC task allows us to leverage AVMIT’s high quality audiovisual annotations whilst effectively multiplying our train set without causing imbalances (many corresponding and non-corresponding samples can be generated for the same visual stream through this shuffling strategy).

AVC tasks provide an interesting test bed for AVMIT; a dataset predicated on having high levels of audiovisual correspondence. Clearly, the “corresponding” samples will be high quality, with the action event confirmed by AVMIT annotations to contain the labelled action. The “non-corresponding” samples, however, may be more limited in AVMIT than in the noisy label case (MIT-16). For instance, MIT-16 will have a broader set of incongruences available during training, which may provide for more robust detection of incongruent samples.

#### DNN architectures

**Fig 7:**
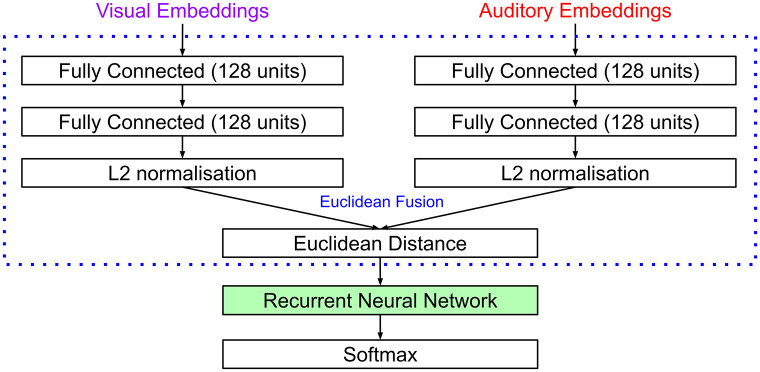
SAVC task architecture. Processing stream for the Supervised Audiovisual Correspondence task. Euclidean distance between audio and visual representations is calculated at each timestep before processing with an RNN and softmax layer.

As in experiment 1, we used either VGGish [20] (audio) and VGG-16 [21] (visual) or YamNet [22] (audio) and EfficientNetB0 [23] (visual) as CNN feature extractors. We found that joining audio and visual feature embeddings at each timestep using our multimodal squeeze unit resulted in poor performance on the SAVC task. We instead employed the audiovisual fusion method of AVE-Net [25], a model developed for the UAVC task. At each timestep, the audio and visual features are individually passed through two 128 unit fully-connected layers (sequentially), before they are L2-normalised and the Euclidean distance is calculated (Fig 7). The Euclidean distance values are passed to either an FRNN, GRU or LSTM before finally a 2-unit softmax layer gives the probability of corresponding/not-corresponding. All RNN models had 256 units and used a dropout rate of 0.1 during training.

#### Training regime

The AVMIT and MIT-16 train sets were further processed as part of the SAVC learning regime. For MIT-16, only audiovisual videos were retained (Fig 4a). Each train set was prepared into 2 parts; the corresponding samples and non-corresponding samples, these were represented equally throughout training to prevent classification biases. The corresponding sample set was formed by pairing each audio stream with a visual stream from any video of the same action class. The non-corresponding sample set was formed by pairing each audio stream with a visual stream of a *different* action class. Many visual stream combinations were used with each audio stream, in both the corresponding and non-corresponding sets. The train set was shuffled and sampled at each epoch.

For each RNN, an instance was trained on this SAVC task using the AVMIT train set, and another identical instance was trained using MIT-16. We use cross-entropy loss and the Adam optimiser [45] with weight decay 10^−5^ and a learning rate of 0.0001 in line with [25]. Although where [25] used a batch size of 2,048, we did not have sufficient resources and so used a batch size of 512 samples. The checkpoint with the best validation accuracy was selected as the final checkpoint for testing.

#### Evaluation and results

We report the cross-entropy loss and binary classification accuracy for the SAVC task. All models trained on AVMIT obtained a lower loss and higher accuracy than their MIT-16 trained counterparts (Table 7). The cleanliness of AVMIT’s “corresponding” class allowed the DNNs to learn a better AVC representation despite the wider range of possible incongruent cases afforded by MIT-16. We further observe that, as in experiment 1, the architectures with older, VGGish+VGG-16, feature extractors performed worse than those with more modern, YamNet+EfficientNet-B0 architectures. While we selected these pretrained feature extractors due to having similar architectures in the audio and visual domains, one may improve performance further by using more modern pretrained feature extractors e.g. [46]. One may further improve performance on the SAVC task by fine-tuning pretrained feature extractors end-to-end, rather than freezing their parameters.

**Table 7:** Supervised Audiovisual Correspondence performance.

Training Set	Embeddings	RNN	Loss	Acc. (%)
AVMIT	YamNet + EffNetB0	FRNN	0.4223	81.30
MIT 16	YamNet + EffNetB0	FRNN	0.5371	73.33
AVMIT	YamNet + EffNetB0	GRU	0.3971	81.82
MIT 16	YamNet + EffNetB0	GRU	0.5124	75.47
AVMIT	YamNet + EffNetB0	LSTM	0.4006	82.24
MIT 16	YamNet + EffNetB0	LSTM	0.5143	74.17
AVMIT	VGGish + VGG-16	FRNN	0.5352	73.18
MIT 16	VGGish + VGG-16	FRNN	0.5695	71.09
AVMIT	VGGish + VGG-16	GRU	0.5289	72.92
MIT 16	VGGish + VGG-16	GRU	0.6921	67.19
AVMIT	VGGish + VGG-16	LSTM	0.4671	77.55
MIT 16	VGGish + VGG-16	LSTM	0.7877	58.39

## Conclusion

We present Audiovisual Moments in Time, a set of audiovisual annotations and DNN embeddings for the Moments in Time dataset. AVMIT contains annotations of 57,177 videos across 41 classes, each pertaining to the existence of an audiovisual event, and its prominence in the video. We demonstrate the utility of AVMIT audiovisual annotations beyond unimodal annotations by training a series of RNNs exclusively on audiovisual data vs. modality-agnostic (audio and/or visual) data and observing an increase of 2.71-5.94% in top 1 accuracy on our audiovisual action recognition task.

We further introduce a new task, the Supervised Audiovisual Correspondence (SAVC) task, whereby a classifier must discern whether audio and visual streams correspond to the same class. This is distinct from previous, unsupervised, AVC tasks whereby a classifier must discern whether audio and visual streams correspond to the same *video*. Importantly in this work, the SAVC task is able to leverage AVMIT’s high quality audiovisual annotations. We use the SAVC task to explore whether AVMIT annotations can be used to explicitly learn more powerful audiovisual representations. We find that training a series of RNNs using AVMIT filtered data improved performance on the SAVC task, with an increase in classification accuracy of 2.09-19.16% vs. unfiltered data.

Alongside AVMIT annotations, we additionally provide a set of 960 videos (60 videos over 16 classes), designated as a controlled test set. These videos can be manipulated for audiovisual synchrony, semantic correspondence, visual or auditory noise etc. to produce a large suite of test videos, suitable for experiments with DNNs and humans alike. Finally, we provide DNN embeddings for AVMIT videos to lower the computational barriers for those who wish to train audiovisual DNNs, thereby levelling the playing field for all. AVMIT provides a useful resource for experiments concerned with audiovisual correspondence, and allows DNN comparisons against humans to take a step into the audiovisual domain.
